# Composition of a Salivary Calculus, Taken from a Horse

**Published:** 1849-01

**Authors:** J. L. Lassigne


COMPOSITION OF A SALIVARY CALCULUS TAKEN FROM A HORSE, by *J. L. Lassigne.*—The calculus was removed from the neck of the first molar tooth of the left upper jaw. In less than a year it had attained the size of a hen's egg: it was of a reddish-white color, remarkably hard, and of a laminated structure. It dissolved in dilute nitric acid with effervescence, and deposited some mucous flocculi. The filtered solution, saturated with ammonia, threw down a white precipitate of phosphate of lime. Analysis of a portion of the calculus furnished the following results:

| | |
|---|---|
| Water, - - - | 3.27 |
| Soluble salivary matter, | 6.19 |
| Mucus, - - - - - | 4.50 |
| Phosphate of lime, - | 2.70 |
| Carbonate of lime, - - | 83.36 |

The essential ingredient in the calculus, therefore, was carbonate of lime, whilst in human salivary calculi, the principal ingredient is phosphate of lime.—*Helleir's Archiv. Sept.* 2, 1846.

